# Occurrence of Deoxynivalenol in Wheat in Slovakia during 2010 and 2011 

**DOI:** 10.3390/toxins5081353

**Published:** 2013-08-02

**Authors:** Svetlana Šliková, Soňa Gavurníková, Valéria Šudyová, Edita Gregová

**Affiliations:** Plant Production Research Centre Piešťany, SK 921 68 Piešťany, Slovakia; E-Mails: gavurnikova@vurv.sk (S.G.); sudyova@vurv.sk (V.Š.); gregova@vurv.sk (E.G.)

**Keywords:** *Fusarium* toxin, *Triticum aestivum* L., produced regions

## Abstract

In this study, a total of 299 grain samples of wheat were collected from four production regions: the maize, sugar beet, potato and feed sectors of Slovakia. The samples were analyzed for deoxynivalenol (DON) content by using an enzyme-linked immunosorbent assay Ridascreen^®^ Fast DON. Analysis of variance revealed a significant difference between years in DON contents (*p* < 0.027). The occurrence of samples with DON was 82.2% in 2010, with maximum DON content of 7.88 mg kg^−1^, and 70.7% in 2011, with maximum DON content of 2.12 mg·kg^−1^. The total mean DON content was 0.62 mg·kg^−1^; in the feed region 0.22 mg·kg^−1^; 0.63 mg·kg^−1^ in the maize region; 0.78 mg·kg^−1^ in the sugar beet region; 0.45 mg·kg^−1^ the potato region. The limit of 1.25 mg·kg^−1^ imposed by the European Union (EU) for DON content was exceeded in 13.7% of the studied samples. The average monthly rainfall for May to June played a critical role in DON content of wheat grains for maize and sugar beet producing regions. The present results indicate that DON content was at a high level in grains from wheat grown during 2010.

## 1. Introduction

The mycotoxin deoxynivalenol (DON) is produced by *Fusarium* species (spp.) which attack wheat during growth and storage if the grain is not sufficiently dried. *F. graminearum* and *F. culmorum* are the main DON-producing fungi in Europe [1]. In the Slovak Republic, the most common species causing Fusarium head blight (FHB) and contamination of wheat grains with mycotoxins are *F. culmorum* (W. G. Smith) Sacc., *F. graminearum* Schwabe [2] and *F. poae* [3]. The occurrence and severity of the disease caused by *Fusarium* spp. is dependent on many factors, which are currently examined in predictive models for FHB and related mycotoxin in wheat [4]. The specificity of FHB lies in the fact that it affects grain formation and that mycotoxins accumulate in infected grains. The grains from the spikes infected by *Fusarium* spp., which are fully developed and without any visible changes, can contain various concentrations of mycotoxins and are especially dangerous. These mycotoxins may contaminate the crop in the initial cereal growing stage in the field and may, moreover, contaminate all stages of the food chain. 

According to the literature, DON is the main *Fusarium* toxic secondary metabolite and its occurrence is also considered to be an indicator of the possible presence of other, more toxic, trichothecenes [5]. The consumption of *Fusarium*-contaminated cereals and cereal-derived products may cause the poisoning known as mycotoxicosis. Several studies have confirmed that the mycotoxin DON, present in animal feed, has toxic effects on animals and similar effects can be expected in humans, e.g., cytotoxic effects that have been detected in human liver cells [6,7]—these report the risks associated with intake of even low levels of DON, but long-term effects in humans have not been documented. 

A worldwide incidence of DON contamination has been reported in cereals [8], in cereal products or feed mixtures (e.g., Europe; [9]). Analyses of cereal products, processed cereal products, as well as wheat-based baby food collected from retailers and farms in Slovakia, have confirmed that of analyzed *Fusarium* mycotoxins, DON was the most prevalent in samples [10]. The toxicity of the mycotoxin has led many countries to set up regulations for its control in grains and food products intended for human or animal consumption. The maximum allowed limits for DON have been set by the European Commission in unprocessed wheat and food products. EC Regulation 1126/2007 [11] applies to unprocessed cereals other than durum wheat and is 1250 µg·kg^−1^. 

The aims of this study were: (1) to provide a picture of the natural occurrence of the mycotoxin DON in wheat grain grown in Slovakia during 2010 to 2011; (2) provide a picture of the natural occurrence of the mycotoxin DON in wheat grown in four producing regions of Slovakia; (3) to investigate the potential for samples with excessive amounts of DON to contaminate primary materials for food production. 

## 2. Results and Discussion

### 2.1. Occurrence of Deoxynivalenol in Naturally Contaminated Wheat Samples during 2010 and 2011

Of the 299 samples analyzed, 76.6% were contaminated with DON (Table 1). In 2010, 82.2% of the samples were contaminated—11.5% more than in 2011. The incidence of DON contamination in 2011 (70.7%) was relatively similar compared to certain reports from other European countries: 65.3% reported in Serbia [12], 65% reported in Croatia [8], and 67% reported in the Czech Republic [13]. Mycotoxin deoxynivalenol is a serious contaminant of cereals and feed, and is dominant in the West and East of Europe. High incidences of DON were found in wheat samples originating from different fields in Austria, Germany, and Slovakia; DON was found in all samples [14]. A similar situation occurred in Italy, when all samples of durum wheat from three areas located in North-Central Italy were found positive for DON in 2010 [15]. Statistically significant differences in the contamination of samples were found between years (Table 2). In our study, markedly higher mean and maximum DON contents were found in samples collected in 2010 compared with 2011 (Table 1). From 2010 to 2011, a 67.7% difference was observed in DON content, with the results showing a high level of sample contamination in 2010. The average contamination of samples in 2010 is the highest detected during our monitoring of the DON content in wheat samples when compared to existing published results [3]. In the samples of wheat grown between 2004 and 2008 collected from nine sites in Slovakia, the analysis found that the highest average contamination was 0.82 mg·kg^−1^, in 2006 [3]. A different DON content was detected in wheat samples collected immediately after harvest, from producers in Serbia in 2004, and had an average contamination of 1.23 mg·kg^−1^. However, between 2005 and 2007 it was substantially lower—at 0.19 mg·kg^−1^ [5]. The range of contamination of the analyzed samples of 2011 (Table 1) was similar to the results presented by Ostrý *et al.* [16], where DON was determined in samples of winter wheat in the Czech Republic at 0.25–3.50 mg·kg^−1^, and 0.00–3.60 mg·kg^−1^ in Romania [17]. 

**Table 1:** Natural occurrence of DON content in wheat samples by years.

Year	No. of samples total/positives ^a^	DON content of samples (mg·kg^−1^)		
		Average	Maximum	Median
2010	152/125	0.93	7.88	0.44
2011	147/104	0.30	2.12	0.20
Total	299/229	0.62	7.88	0.20

^a^ Positive samples: mycotoxin concentration above detection limit >0.2 mg·kg^−1^.

**Table 2:** ANOVA for deoxynivalenol (DON) content in wheat samples collected from Slovakia during 2010 and 2011.

Source of variation	Sum of squares	Degree of freedom	Mean square	*F*-Value	*p*-Value
Year	30.08	1	30.08	29.84	0.00
Error	299.44	297	1.01		
Total	444.62	299			

Contamination with DON is affected by environmental conditions. Research has showed [18] that season-by-season conditions are the most significant factor, which has up to a 48% influence in the production of DON. A positive relationship between rainfall and wheat grain contamination with mycotoxins was revealed—during years with higher rainfall, the grain contamination in wheat grown in nine localities of Slovakia increased. It showed a significant influence of climatic conditions for DON contamination [19]. Our data from the weather stations (WS) showed that the average rainfall was higher in during May and June 2010, when wheat was—according to phenological observations—in the flowering stage or after flowering, than in 2011 (Table 3). A positive correlation was found between the amount of rainfall and DON content in May and June (Table 4). Van der Fels-Klerx *et al*. [20] reported that relative humidity during wheat cultivation, particularly in the period around flowering, increased DON concentrations at wheat harvest. A direct relationship between contamination of wheat with DON has been established. Vančo *et al*. [21] reported that a positive correlation was found between the amount of DON in grains of wheat harvested in the 2004 season in Slovakia` and the amount of rainfall in June. The incidence of FHB is strongly associated with moisture at the time of flowering (anthesis).

**Table 3:** The rainfall and temperature in Slovakia in May and June 2010–2011.

	Produced Regions	May	May	June	June
		2010	2011	2010	2011
Rainfall (mm)	PFR	196	67	127	120
	PMaR	168	45	127	84
	PSBR	149	61	102	91
	PPR	169	71	171	103
	Sum	682	244	527	398
Temperature (°C)	PFR	12.3	13.0	16.5	16.3
	PMaR	15.2	16.1	19.6	19.9
	PSBR	14.8	15.3	19.2	18.8
	PPR	13.2	13.3	17.2	17.1
	Mean	13.86	14.43	18.13	18.03

Abbreviations: PFR—produced feed; PMaR—produced maize region; PSBR—produced sugar beat region; PPR—produced potato region.

**Table 4:** Correlation coefficients by Pearson.

	Total rainfall/DON		Average temperature/DON	
	May	June	May	June
Pearson coefficient	0.284	0.191	−0.052	0.054
*p*-Value	0.000	0.001	0.372	0.356

### 2.2. Occurrence of Deoxynivalenol in Wheat Samples of Producing Regions

The results presented in Table 4 show the natural occurrence of DON in wheat samples grown in producing regions of Slovakia (PFR—produced feed; PMaR—produced maize region; PSBR—produced sugar beat region; PPR—produced potato region). On average, the highest percentage of positive samples was found in samples from PPR and the lowest in the PFR in 2010 and 2011. Differences between 2010 and 2011 in percentage of positive samples were the most significant in PMaR. In 2010, the highest percentage of these samples was found in this area. In 2010, 3.7 times greater rainfall fell in May and 1.5 times greater fell in June than in 2011 during the flowering and post-flowering periods of wheat. These results agree with those described by Hooker *et al*. [22], that DON content in grains is highly associated with weather conditions at flowering and after flowering. The average DON content of samples in the PFR for 2010 and 2011 was the lowest. The high rainfall, but the lowest mean temperatures, were in PFR in both years (Table 3). The mean low level contamination might be associated with low temperature (Table 5). This fact agrees with arguments by Hooker *et al.* [22], which reported that DON content was positively correlated with rainfall and was decreased with air temperatures below 10 °C during the period before heading, with the timing of the rainfall being more influential than the amount of precipitation. In 2011, only small differences in the average DON contamination were apparent between production areas. The greatest difference—of 19.4%—was between PSBR and PPR. In 2010, the differences between contamination was greater, especially between PSBR and PFR (54.9%). In PMAR, the average contamination of samples in 2010 was 3.3 times higher than in 2011. However, the highest differences were found in PSBR, when sample contamination in 2011 was 3.6 times lower than in 2010. Lori *et al*. [23] reported that among five locations, differences in the contamination levels of wheat samples were found and the samples from locations situated in a humid area showed higher levels of DON content than those in dry areas. These results show that the rainfall and temperature were factors influencing contamination of grain wheat. The different level of contamination between years was more affected by rainfall than temperature. The different level of contamination between produced regions was more affected by temperature than rainfall in some year.

**Table 5:** Natural occurrence of DON content in wheat samples from produced regions in years 2010 and 2011.

Year	Produced region	No. of samples total/positives ^a ^ —Percentage	DON content of samples (mg·kg^−1^)	
			Average	Max.
2010	PFR ^b^	6/3—50%	0.12	0.32
	PMaR ^c^	108/101—93.5%	0.96	7.88
	PSBR ^d^	20/8—40%	1.31	5.80
	PPR ^e^	18/13—72.2%	0.59	1.42
2011	PFR ^b^	6/4—66.6%	0.33	1.36
	PMaR ^c^	104/69—66.3%	0.29	2.12
	PSBR ^d^	26/20—76.9%	0.36	1.58
	PPR ^e^	11/11—100%	0.31	0.78
Mean 2010 and 2011	PFR ^b^	12/7—58.3%	0.22	1.36
	PMaR ^c^	212/170—80.2%	0.63	7.88
	PSBR ^d^	46/28—60.9%	0.78	5.80
	PPR ^e^	29/24—82.7%	0.45	1.42

**Abbreviations: **^a^ Positive samples: mycotoxin concentration above detection limit > 0.2 mg kg^−1^; ^b^ PFR—produced feed; ^c^ PMaR—produced maize region; ^d^ PSBR—produced sugar beat region; ^e^ PPR—produced potato region.

### 2.3. Occurrence of Samples with Excessive Amounts of DON

The occurrence of samples with excessive amounts of DON is rare, but such samples are dangerous for processors and direct consumers, as the cereal products and food products from flour are heavily contaminated. The limit for DON content determined by the European Union (1.25 mg·kg^−1^) was exceeded in 13.7% of the analyzed samples. This result is similar to a report from Romania, where the limit was exceeded in 14% of wheat samples [17]. In 2010, 21.7% of samples (*N* = 33/152) were contaminated with DON ranging from 1.25 to 7.88 mg·kg^−1^. In 2011, samples contained 4.6 times lower than 2010 (*N* = 8/147). The maximum DON content (7.88 mg·kg^−1^) was found in one sample obtained in 2010 originating from PMaR (Table 5). In Poland, there were occurrences of FHB of wheat in the 2009 season and DON content was estimated to be between 1.7 and 11.9 mg·kg^−1^ for healthy looking grains [24]. The limit was exceeded in 8.3% of the samples from the feed producing region, 13.7% from the maize producing region, 19.6% from the sugar beet producing region, and 6.9% from the potato producing region (Table 6). Monitoring of samples from 2004 to 2006 in Slovakia revealed statistical differences in the average contamination between production areas [19]. By monitoring the occurrence of DON in wheat grown in Slovakia from 2004 to 2006, the highest percentage of samples (14.3%) that exceeded the limit occurred in potato producing region [19], a lower incidence (9.3%) was in maize producing region, and from sugar beet region it was 5% of the samples. Researchers indicated that rainfall in May from 2004 to 2006 was the heaviest (136 mm) in the potato producing region. This amount of rainfall was much lower when compared to 2010 (Table 3). Limits were exceeded in the PMaR and PSBR for DON content in both years and the highest percentages of samples exceeding the permissible limit of EU were in the PSBR (Table 6). It is difficult to infer trends or recent developments regarding high DON contamination in grains because the occurrence of contaminated samples is influenced by many factors [25]. Contamination levels higher than those allowed might be associated with factors other than climate conditions—known to be due to mycotoxin formation, e.g., heavy rainfall before harvest [26], crop rotation (maize as a pre-crop for wheat) and growing highly susceptible wheat cultivar if no fungicide is applied [27]).

**Table 6:** The distribution of the DON contents in wheat samples by produced regions.

DONcontent of samples (mg·kg^−1^)	No. of samples/%								
	PFR		PMaR		PSBR		PPR		**Total**
	2010	2011	2010	2011	2010	2011	2010	2011	
<0.20	3/50%	2/33.3%	7/6.5%	35/33.6%	12/60%	6/23.1%	1/5.6%	4/36.4%	71/23.8%
0.20–1.25	3/50%	3/50%	75/69.4%	66/63.5%	3/15%	16/61.5%	15/83.3%	7/63.6%	187/62.5%
>1.25	0/0%	1/16.7%	26/24.1%	3/2.9%	5/25%	4/15.4%	2/11.1%	0/0%	41/13.7%
Total	6	6	108	104	20	26	18	11	299
Total	12		212		46		29		299

Abbreviations: PFR: produced feed; PMaR: produced maize region; PSBR: produced sugar beat region; PPR: produced potato region; 2010 and 2011: years in which measurement was taken.

## 3. Experimental Section

In 2010 and 2011, mature wheat grain were collected from 169 fields in Slovakia (in the 2010 season from 100 fields and in the 2011 season from 69 fields) for the monitoring of DON content. Each wheat sample was collected from a different field situated in one of the four produced regions, broken down topologically as follows, PFR: elevation above sea level (EASL) >600 m, average annual temperature: 6 °C, annual rainfall above >800 mm, the region is characterized podzolic and feed land; PMaR: EASL 112–200 m, average annual temperature 9–10 °C; annual rainfall >600 mm, land type of black soil and, in particular, brown soil; PSBR: EASL 200–350 m, average annual temperature 8–9 °C, annual rainfall >600–700 mm, land type consisting of brown soil to clay-brown soil; PPR: EASL 350–600 m, average annual temperature 7–8 °C, annual rainfall >700–800 mm, land type consisting of brown soil to clay-brown soil.

The data relating to precipitation and temperature are from WS from the PFR (Poprad, Banská Štiavnica, and Liptovský Hrádok), the PMaR (Hurbanovo, Mochovce, Piešťany, Podhájska, and Žihárec), PSBR (Beluša, Prievidza, Trenčín) and the PPR (Turčianske Teplice, Spišské Vlachy, and Vígľaš); all locations are located in Slovakia. 

Approximately 1 kg of grain samples sent by farmers to the Institute of Plant Production (Piešťany, Slovakia) immediately after the harvest were stored in refrigerated conditions below 5 °C. A commercial ELISA kit was used to determine the DON concentration in wheat samples (Ridascreen^®^ Fast DON; RBiopharm, Darmstadt, Germany). The grain samples were ground (Ultra Centrifugal Mill, type ZK 100; Retsch, Haan, Germany), and subsequently 100 mL of distilled water was added to 5 g of each sample and the mixture filtered. Aliquots of 50 µL of filtrate were used for analysis. The absorbancies (of the wells) were determined photometrically at 450 nm (MRX II; Dynex Technologies, Chantilly, VA, USA). DON concentrations were calculated in mg·kg^−1^ by Revelation Version 4.25 (Dynex Technologies). Statistical analysis was performed using SPSS software 11.5 (SPSS, Chicago, IL, USA), with a statistical significance set at 95% (*p* < 0.05) and 99% (*p* < 0.01).

## 4. Conclusions

By analyzing the DON content in the samples of wheat grown in different localities in Slovakia between 2010 and 2011, it was found that the grain harvested in 2010 was often highly contaminated. A relationship was confirmed between the occurrence and levels of mycotoxins and heavy rainfall in May and June in Slovakia, when the right conditions for the development of DON-producing *Fusarium* were created. The highest percentage of contaminated samples came from sugar beet producing region in both years. In 2010, a high occurrence of samples exceeding the EU regulation limit established for unprocessed grain was seen. The presence of such samples is often connected with multi-toxin contamination of grains, as well as the synergistic toxicity of DON with other mycotoxins. 
